# Validierung eines neuen portablen Messsystems für die diagnostische Heimnutzung – eine Machbarkeitsstudie

**DOI:** 10.1007/s00120-026-02855-y

**Published:** 2026-05-27

**Authors:** Ulrike Wirth, Sigrun Holze, Christian Wagner, Alena Bonaventura, Stefan Siemer

**Affiliations:** 1 Klinik für Urologie Universitätsklinikum Leipzig, Liebigstraß 20, 04103 Leipzig, Deutschland; 2 Klinik für Urologie, Urologische Onkologie und Roboter-assistierte Chirurgie, Möllenweg 22, 48599 Gronau, Deutschland; 3 Klinik und Poliklinik für Urologie und Kinderurologie, Universitätsklinikum des Saarlandes, Kirrbergerstraße 100, 66421 Homburg, Deutschland

**Keywords:** Heimgebrauch, Uroflow, Biomarker, International Prostate Symptom Score, Streamcheck®, Home use, Uroflow, Biomarkers, International Prostate Symptom Score, Streamcheck®

## Abstract

**Hintergrund:**

Zu den häufigsten Erkrankungen bei Männern gehören LUTS, Nieren- und Blasenerkrankungen. Da Männer trotz zahlreicher Aufklärungsmaßnahmen nicht an Vorsorgeuntersuchungen bzw. Früherkennungsmaßnahmen teilnehmen, soll in der aktuellen Studie überprüft werden, ob Männer auch in der häuslichen Umgebung Früherkennungsmaßnahmen durchführen können. Da insbesondere Männer durch digitale Medien angesprochen werden, wurde ein digitales Messinstrument für den Heimgebrauch entwickelt (Streamcheck®, [SC]), das wichtige Abklärungsmaßnahmen wie Uroflow, Biomarker und Standardfragebögen enthält.

**Ziel der Arbeit:**

Das Ziel der Arbeit war, SC in einer klinischen Untersuchung unter häuslichen Bedingungen zu überprüfen.

**Material und Methoden:**

Eingeschlossen und mit dem SC untersucht wurden 92 Männer zwischen 23 und 89 Jahren. Die Aufzeichnung der Urinflusskurve, das Urinvolumen, die Miktionszeit als auch die Biomarker wurden bestimmt. Die Patientenzufriedenheit wurde an Hand von Fragebögen überprüft.

**Ergebnisse:**

Die Befunde von 73 Patienten konnten ausgewertet werden. Bei allen Männern wurde eine Uroflowkurve dargestellt mit vergleichbarem Urinvolumen (Mittelwerte: SC = 360 ml, Kontrolle = 364 ml) und Miktionszeit (maximaler Unterschied 4,8 %; n. s.). Die Biomarker wurden mit einer 100 %-Sensitivität und > 95 %-Spezifität erkannt. Die Männer waren im Mittel mit 4,04 Punkten (1 = sehr unzufrieden; 5 = sehr zufrieden) mit dem SC zufrieden.

**Schlussfolgerung:**

Der SC bestätigt sich als zuverlässige Messmethode für den Heimgebrauch bei hoher Patientenzufriedenheit. Inwieweit die Ergebnisse das Patientenverhalten im Hinblick auf eine Arztvorstellung beeinflusst, wird in einer nachfolgenden Studie untersucht.

## Abstract (en)

**Background:**

Lower urinary tract symptoms (LUTS), kidney, and bladder diseases are among the most common diseases in men. Despite numerous educational measures, men do not participate in preventive medical checkups or early detection measures. The current study aims to examine whether men can also carry out early detection measures in their home environment. Since men are particularly receptive to digital media, a digital measuring device for home use (the Streamcheck®, SC; Vierkirchen, Germany) was developed that includes important diagnostic measures such as uroflow, biomarkers, and standard questionnaires.

**Objective:**

The aim of the study was to evaluate SC in a clinical trial under home conditions.

**Materials and methods:**

A total of 92 men between the ages of 23 and 89 were included and examined with the SC. The urine flow curve, urine volume, micturition time, and biomarkers were recorded. Patient satisfaction was assessed using questionnaires.

**Results:**

The findings of 73 patients were evaluated. A urine flow curve was obtained for all men with comparable urine volumes (mean: SC = 360 ml, control = 364 ml) and micturition time (maximum difference 4.8%; n. s.). The biomarkers were detected with 100% sensitivity and >95% specificity. The men were very satisfied with the SC, with an average score of 4.04 points (1 = dissatisfied, 5 = very satisfied).

**Conclusion:**

The SC has been confirmed as a reliable measurement method for home use with high patient satisfaction. The extent to which the results influence patient behavior with regard to consulting a physician will be investigated in a subsequent study.

Seit Jahren versuchen Versicherungen, Krankenkassen und u. a. die DGU Männer vermehrt zu Vorsorge‑/Früherkennungsprogrammen zu motivieren, ohne größeren Erfolg. Anstatt Männer zum Arzt zu bewegen, soll mit einer neuen Untersuchungsmethode, dem Streamcheck® (SC, Streamcheck GmbH), Männern die Möglichkeit gegeben werden, von zu Hause aus unterschiedliche Früherkennungsuntersuchungen durchzuführen. Die KI-unterstützte Auswertung empfiehlt bei auffälligen Befunden einen Arztbesuch. Vergleichbare Ansätze werden in den USA und z. B. in den Niederlanden beobachtet.

## Hintergrund und Fragestellung

Eine der häufigsten Erkrankungen bei Männern mit zunehmendem Alter sind Symptome des unteren Harntrakts (LUTS), wobei weltweit bis zu 2,3 Mrd. Menschen mit LUTS-Beschwerden beschrieben werden [2, 12]. Zur Abklärung möglicher Ursachen von Miktionsstörungen können nicht-invasive und invasive diagnostische Verfahren zur Anwendung kommen. Die Uroflowmetrie, eine Urinanalyse und der Internationale Prostata-Symptom-Score (IPSS) sind häufig angewandte nicht-invasive Untersuchungsmethoden [5, 15]. Die Uroflowmetrie wird als einer der wichtigsten primären Untersuchungsmethoden bei LUTS Beschwerden von der European Association of Urology (EAU), der International Consultation on Incontinence (ICI) und der American Urological Association (AUA) empfohlen [1, 12, 14, 21]. Da diese Untersuchung aber sehr zeitaufwendig ist, Personal bindet und schlecht vergütet wird, erfolgt die Uroflowmetrie in deutschen Kliniken/Praxen nur selten.

Neben der Uroflowmetrie wird im Rahmen einer routinemäßigen Gesundheitsabklärung häufig eine Urinanalyse angeboten. Während im 20. Jahrhundert die Urinsedimentanalyse den Goldstandard darstellte, haben sich Urinteststreifen aufgrund der besseren Verfügbarkeit und der geringeren Kosten zunehmend durchgesetzt [6, 9, 16].

Aber auch wenn Uroflowmetrie, Urinteststreifen und IPSS-Fragebögen einfach in der Handhabung sind, werden sie von Männern nur selten angewandt. Dies liegt v. a. daran, dass Männer nur selten zur Vorsorgeuntersuchung bzw. Früherkennung zum Arzt gehen. Woran liegt das? Wartezeiten beim Arzt werden von Männern deutlich schlechter akzeptiert, zeitaufwendige Untersuchungen häufig als nicht notwendig angesehen. Die Digitalisierung der Medizin (u. a. Apps) spricht hingegen Männer deutlich mehr an. Durch zahlreiche Kampagnen der Krankenkassen und Versicherungsträger und der DGU (u. a. Urologische Stiftung Gesundheit: „Prostata – mach es zu deinem Bier“) konnte die Rate an Männern, die zur Vorsorge/Früherkennung erschienen, jedoch nicht deutlich gesteigert werden. Alle diese Maßnahmen hatten eines gemeinsam: Männer zur Vorstellung bei einem Urologen zu bewegen.

### Wenn Männer nicht zum Arzt gehen, erfolgt die Abklärung zu Hause

Daher scheint die Annahme logisch: Wenn Männer nicht zum Arzt gehen, müssen relevante Untersuchungen zu Hause, im geschützten Umfeld durchgeführt werden können, und nur bei Auffälligkeiten sollte der Arzt/Urologen aufgesucht werden.

In der aktuellen Studie wird ein neues Untersuchungsmodul (SC) für den Heimgebrauch bei Patienten getestet, um die Akzeptanz der Männer für Früherkennungsmaßnahmen zu verbessern. In der vorliegenden Arbeit soll überprüft werden, ob mit dem System eine Harnstrahlkurve aufgezeichnet und die Urinmenge korrekt beschrieben wird. Zudem sollen neben der Miktionszeit verschiedene Biomarker mit herkömmlichen Messmethoden überprüft werden. Zudem sollen der Patientenkomfort und die Patientenzufriedenheit abgefragt werden.

## Studiendesign und Untersuchungsmethoden

**Abb. 1:**
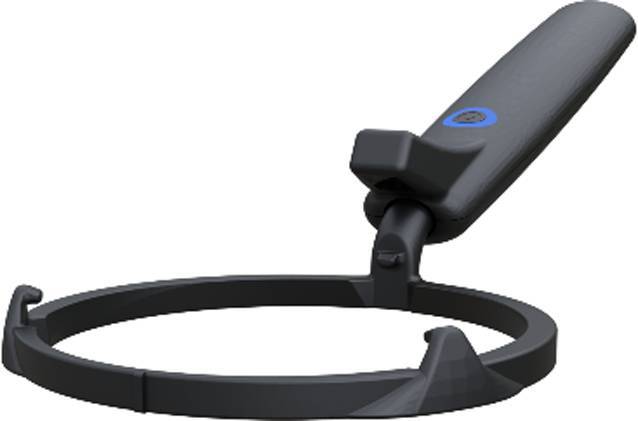
Streamcheck®-Device mit Haltegriff und integrierter Kamera

**Abb. 2:**
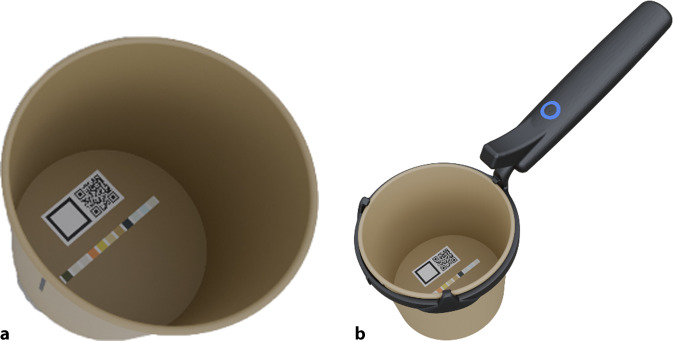
**a** Streamcheck®-Becher mit Biomarkerteststreifen (Blut, Protein, Leukozyten, Nitrit, pH) und QR-Code. **b** Streamcheck® mit einliegendem Becher bereit für die Messung

**Abb. 3:**
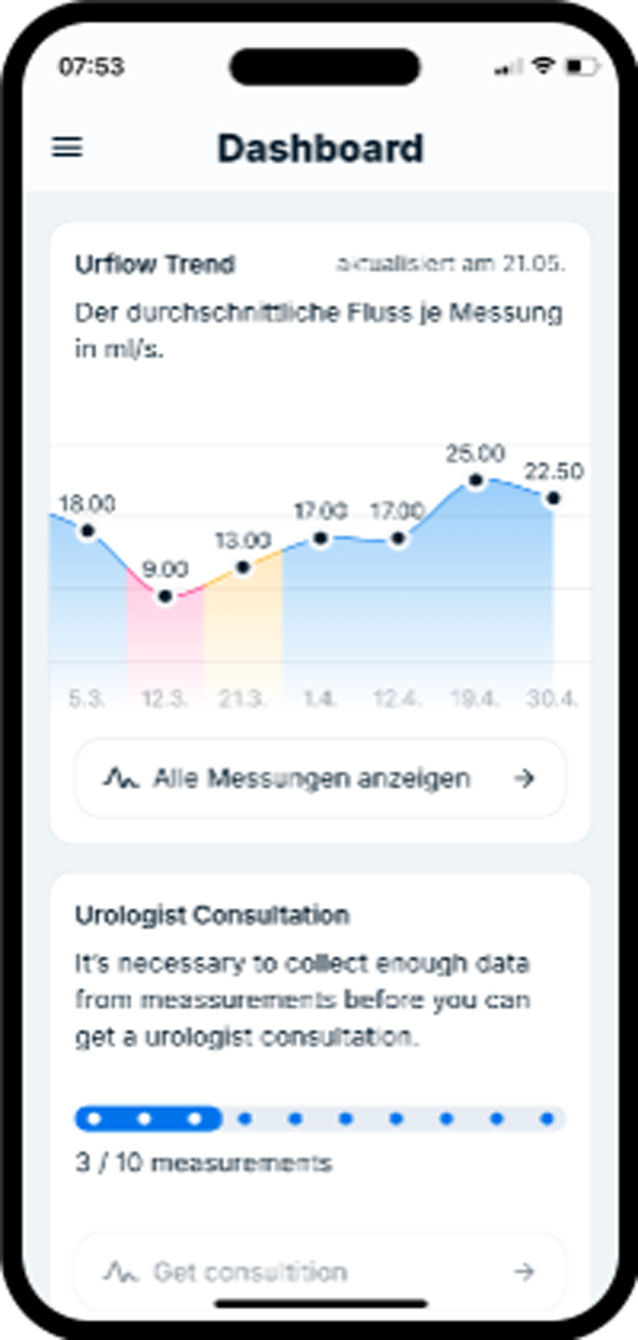
Streamcheck®-Dashboard mit der Darstellung für den Patienten

Der SC ist ein Medizinprodukt der Klasse 1M. Er besteht aus einem Handgriff mit Kamera und Messeinheit (Abb. 1). Der Messbecher, der den Teststreifen für die Biomarker im Urin (Blut, Protein, Leukozyten, Nitrit, pH) und einen QR-Kode enthält (Identifizierung des Patienten, Abb. 2) wird in den dafür vorgesehenen Ring eingelegt. Anschließend miktioniert der Patient in den Becher. Der Uroflow wird erfasst und via Bluetooth mit dem Patientenhandy verbunden. Daten und Flusskurve werden in der App (Abb. 3) gespeichert. Der Becher wird entleert und die Biomarker werden von der Kamera ausgelesen. Die Auswertung findet KI-basiert statt und wird in der App zur Verfügung gestellt. Es werden Handlungsempfehlungen nach einer programmierten Medizinlogik angezeigt. Über das Dashboard werden eine Trendanalyse aller gemessenen Werte sowie das Datum der nächsten Planmessung angezeigt. Die Messergebnisse werden in Berichtsform dem Facharzt zur Verfügung gestellt. Der Facharzt kann dem Patienten zum nächsten Plantermin über die App eine Benachrichtigung als Erinnerung schicken (E-Mail/Benachrichtigung). Bei Auffälligkeiten der Werte wird eine Kontaktaufnahme mit dem Facharzt vorgeschlagen, so dass eine zeitnahe Konsultation erfolgen kann.

Für die Zertifizierung wurde im Labor die korrekte Ableitung der Uroflowkurve in standardisierten Untersuchungen nachgewiesen. In der hier vorliegenden ersten, prospektiv klinischen Studie soll die Patientenanwendung unter reellen Bedingungen (simulierte häusliche Umgebung) untersucht werden. Hierbei soll das Vorhandensein einer aufgezeichneten Uroflowkurve, das Miktionsvolumen, die Miktionszeit und die korrekte Darstellung der Biomarker im Vergleich zu herkömmlichen Messmethoden überprüft werden. Zudem wurde die Patientenzufriedenheit anhand von Fragebögen untersucht.

Die Stichprobengröße für die Hauptstudie wurde anhand statistischer Berechnungen ermittelt, um die Zuverlässigkeit und Repräsentativität der Ergebnisse zu gewährleisten. Dabei wurden folgende Parameter verwendet: Populationsgröße (*N*): 1000, Konfidenzniveau (t): 90 %, entsprechend einem t‑Wert von 1,65. Erwarteter Anteil (*p*): 50 % (*p* = 0,5), was der konservativsten Annahme entspricht. Fehlerspanne (d): 10 % (d = 0,1), was für ein Medizinprodukt mit geringem Risiko akzeptabel ist. Die Stichprobengröße wurde mit der folgenden Formel berechnet:

$$n=\frac{N\cdot t^{2}\cdot p\cdot \left(1-p\right)}{d^{2}\cdot (N-1)+t^{2}\cdot p\cdot (1-p)}$$

Einsetzen der Werte:

$$\begin{aligned}&n=\\&\frac{1000\cdot 1{,}65^{2}\cdot 0{,}5\cdot \left(1-0{,}5\right)}{0{,}1^{2}\cdot (1000-1)+1{,}65^{2}\cdot 0{,}5\cdot (1-0{,}5)}\\&=64\end{aligned}$$

Die Berechnung ergibt eine erforderliche Mindeststichprobengröße von *n* = 64 Teilnehmern. Um möglichen Datenverlusten oder Protokollabweichungen Rechnung zu tragen, erhöhte das Studienteam die Stichprobengröße auf 92 Teilnehmer. Diese Anpassung gewährleistet eine ausreichende Datenqualität und Robustheit bei gleichzeitiger Wahrung der praktischen Umsetzbarkeit. Die ausgewählte Stichprobengröße ist statistisch gültig und bietet ein angemessenes Gleichgewicht zwischen Präzision und Ressourcenzuweisung für die beabsichtigte Bewertung des SC-Geräts.

Das Patientenalter lag zwischen 23 und 89 (Durchschnittsalter 55,78) Jahren, was die vorgesehene Anwendergruppe repräsentiert. Von den 92 initialen Messungen des Urinvolumens wurden 14 Messungen ausgeschlossen, da das Miktionsvolumen < 50 ml und damit außerhalb des zuverlässigen Grenzbereichs eines Uroflows lag. 5 weitere Messungen wurden ausgeschlossen, da die Teilnehmer ihren Penis während der Miktion kurz auf die Halterung für den Becher ablegten, was die Ergebnisse möglicherweise verfälschte. Insgesamt gingen 73 Messungen in die abschließende Bewertung ein.

Die primären Endpunkte der Studie waren:

1. Genauigkeit der Urinvolumenmessung in der Patientenanwendung (± 10 % Abweichung von der Kontrolle),
2. Übereinstimmung der Biomarkerbestimmung mit der visuellen Streifenauswertung.

Sekundäre Endpunkte:

1. Plausibilität der Uroflowkurven basierend auf Expertenbewertung,
2. Überprüfung der Miktionszeit (± 10 % Abweichung von der Kontrolle),
3. Patientenzufriedenheit.

Ein entsprechendes Ethikvotum für diese Studie liegt vor.

## Ergebnisse

Die Studie wurde über einen Zeitraum von 4 Wochen durchgeführt und umfasste die Teilnehmerrekrutierung, Datenerhebung und -analyse.

Urinvolumenmessung: Patienten miktionieren in den Becher. Das Miktionsvolumen wird in einer App aufgezeichnet und dokumentiert. Nach Abschluss der Miktion wird der Urin in einen Messbecher überführt und abgemessen. Dieser Wert gilt als Kontrolle für die Messung des Urinvolumens mit dem SC-System. Urinvolumina < 50 ml wurden wie oben beschrieben nicht berücksichtigt, da hierbei die Bestimmung des Uroflows nicht ausreichend aussagekräftig ist.

**Abb. 4:**
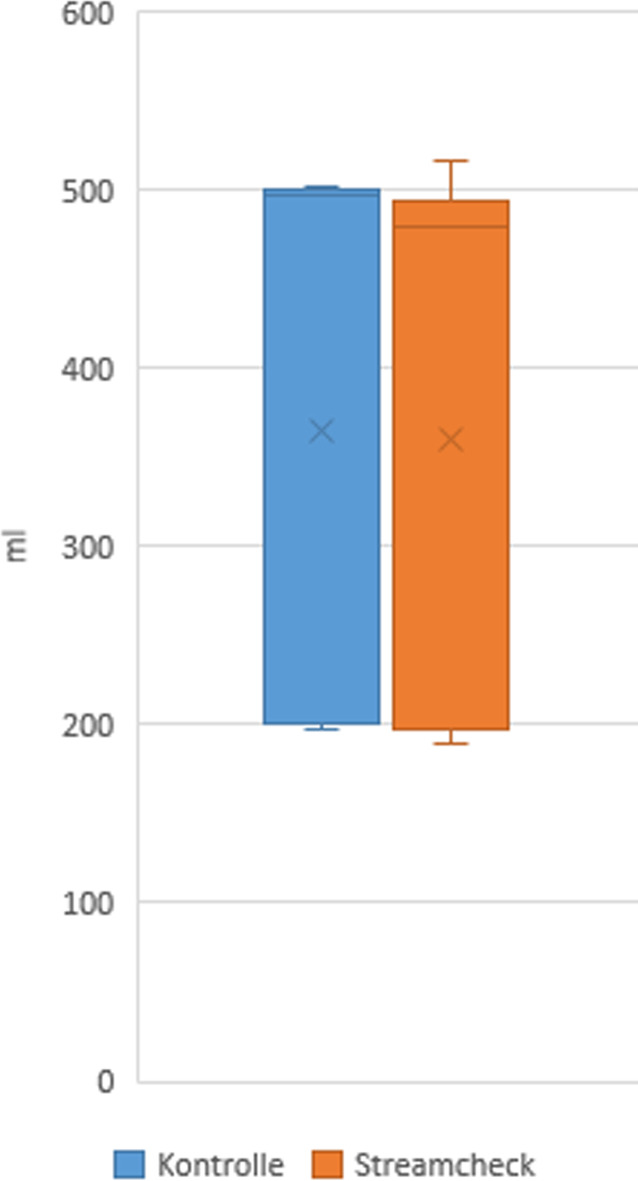
Bestimmung des Miktionsvolumens mit Streamcheck®. Mittel: 360 ml (Standardabweichung [SD]: 148) Kontrolle: 364 ml (SD: 149, Abweichung < 10 %)

Das Miktionsvolumen in der Kontrolle lag im Mittel bei 364 (± 149) ml, in der SC-Messung bei 360 (± 148) ml und lag somit deutlich unter der 10 % erlaubter Abweichung. Der mittlere absolute prozentuale Fehler (MAPE) betrug bei der Urinvolumenbestimmung 2,73 %. Alle Messungen lagen innerhalb des definierten Akzeptanzbereichs von ± 10 %, was die Genauigkeit der Volumenmessung bestätigt (Abb. 4).

### Uroflowparameter vom SC vergleichbar mit Kontrollen

**Abb. 5:**
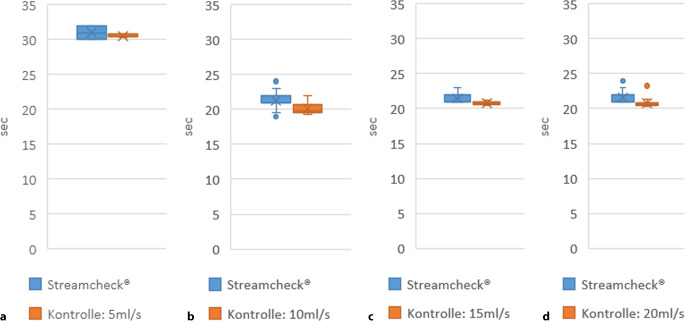
Miktionszeit (Labor) unter definierten Bedingungen mit jeweils *n* = 15 Messungen: Mittelwerte in s (Standardabw.): **a** SC 31 (± 0,73); K: 30,51 (± 0,19); **b** SC 21,24 (± 1,15); K: 20,16 ± (0,80); **c** SC 21,4 (± 0,61); K: 20,7 (± 0,25); **d** SC 21,6 (± 0,87); K: 20,76 (± 0,71); **a**–**d** (Abweichung < 10 %)

Miktionszeit: Die Miktionszeit wurde unter Laborbedingungen simuliert. Es wurde ein definierter Wasserstrahl über eine definierte Zeit aufgebaut und mit dem Streamcheck® gemessen. Es sollte hierbei die Zeit, die der Wasserstrahl in den Becher läuft bei unterschiedlichen Bedingungen (5–20 ml/s; Volumen 150–400 ml) gemessen werden (Abb. 5). Die maximale Abweichungstoleranz wurde mit 10 % festgelegt. Jeder Messpunkt wurde 15-mal wiederholt. Es kamen unterschiedliche SC-Geräte zum Einsatz.

Unabhängig vom Urinfluss (5–20 ml/s) und unabhängig vom Volumen (150–400 ml) waren die Messungen in der Kontrollgruppe zu den Messungen mit dem SC-System und in der App aufgezeichneten Werte nicht signifikant unterschiedlich (Abb. 5; *p* > 0,5). Der größte Unterschied mit 4,8 % wurde bei einem Fluss von 10 ml/s über ca. 20 s (Volumen ca. 200 ml) beobachtet und lag deutlich unter der Abweichungstoleranz von 10 %.

Plausibilität der Uroflowkurve: Expertenbewertungen (Urologen) bestätigten, dass 100 % der von den Patienten gemessenen Uroflowkurven klinisch plausibel waren und keine Artefakte oder Unregelmäßigkeiten aufwiesen. In einigen Kurven wurden vereinzelt Flusskurvenspitzen beobachtet, die durch vorübergehenden äußeren Druck aufgrund der kurzzeitigen Platzierung des Penis auf dem Cup-Ring erklärt werden konnten. Diese artefaktbehafteten Messungen/Patienten (*n* = 5) wurden von der Studie ausgeschlossen.

Biomarkererkennung: Das Gerät erkannte Biomarker mit einer Gesamtgenauigkeit von 98,7 % und erreichte damit die vordefinierte Akzeptanzschwelle von ≥ 90 %. Die Sensitivität wurde mit 100 %, die Spezifität mit ≥ 95 % für alle Biomarker bestimmt. Der positive Vorhersagewert (PPV) lag bei ≥ 85 % für die meisten Biomarker (außer Nitrit mit 50 % [geringe Anzahl positiver Ergebnisse]). Der negative Vorhersagewert (NPV) wurde mit 100 % für alle Biomarker festgehalten. Bei der pH-Wert-Messung zeigte das SC-System eine mittlere Differenz von +0,02 und einen Pearson-Korrelationskoeffizienten von 0,99, was auf eine ausgezeichnete Übereinstimmung mit den Referenzwerten hindeutet.

### Patientenzufriedenheit mit SC hoch

Die Benutzerfreundlichkeit wurde anhand von 11 Fragen überprüft: 48/73 (66 %) Teilnehmer beantworteten alle Fragen und konnten für die finale Auswertung berücksichtigt werden. Auf einer Skala von 1 (sehr unzufrieden) bis 5 (sehr zufrieden) wurde durchschnittlich eine Bewertung für die Benutzerfreundlichkeit von 4,04 angegeben. 79,2 % (38/48) der Patienten waren mit dem Gerät „zufrieden“ oder „sehr zufrieden“. Kein Teilnehmer äußerte Unzufriedenheit (Bewertung ≤ 2). Die durchschnittliche Bewertung für die Verständlichkeit der Anleitung betrug 3,96. 31,3 % (15/48) bewerteten die Verständlichkeit der Anleitung als neutral. Kein Teilnehmer äußerte Unzufriedenheit (Bewertung ≤ 2). Die durchschnittliche Bewertung für die *Gesamtzufriedenheit *betrug 3,98. 68,8 % (33/48) sind mit dem Gerät „zufrieden“ oder „sehr zufrieden“. Kein Teilnehmer äußerte Unzufriedenheit.

Zusammenfassend wurden sowohl die primären als auch sekundären Endpunkte erreicht.

## Diskussion

Zahlreiche epidemiologische Arbeiten bestätigen seit langem konsistent, dass sich Männer und Frauen in Bezug auf Gesundheit, Krankheiten aber auch v. a. in gesundheitsrelevanten Verhaltensweisen und der Inanspruchnahme von Versorgungsleistungen deutlich unterscheiden [10, 17–20]. Während fast 70 % der Frauen das Angebot einer Krebsvorsorge annehmen, wird nicht einmal jeder 2. Mann zur Krebsvorsorge vorstellig (Robert Koch Institut, RKI). Nach der „Studie zur Gesundheit Erwachsener in Deutschland“ (DEGS) des RKI wurde lediglich bei 38,9 % der anspruchsberechtigten Männer ab dem 45. Lebensjahr innerhalb von 12 Monaten vor der Befragung eine Abklärung der Prostata durchgeführt [20]. Als einer der Gründe für die deutlich bessere Akzeptanz bei Frauen wird der Erstkontakt bereits im jugendlichen Alter angesehen. Die jungen Frauen werden hier meist mit der/durch die Mutter beim Arzt vorgestellt. Die meisten Angebotsstrukturen sind jedoch nicht geschlechterspezifisch. Sie erreichen die Männer nur deutlich schlechter. Wenn nichts weh tut, geht „Mann“ nur selten zum Arzt, sprich zur Vorsorgeuntersuchung/Früherkennungsmaßnahmen, auch wenn ihm ab dem 35 LJ. ein Hautscreeningtest und alle 3 Jahre ein Gesundheitscheckup zusteht [17]. Unterschiedliche Versicherungen (AOK, TK, Barmer) aber auch die Fachgesellschaft (DGU) haben in der Vergangenheit versucht, Männer zu motivieren einen Arzt/Urologen zur Früherkennung/Vorsorge aufzusuchen. Die Rate an Vorsorgeuntersuchungen hat sich dadurch jedoch mit 9–13 % nicht nennenswert verändert (Prostata Hilfe Deutschland Mai 2021). In Österreich nahmen 2022 auch weiterhin nur ca. 12 % der Männer an Vorsorgeuntersuchungen teil (Österreichische Gesundheitskasse 2023).

Aufgrund eines komplett unterschiedlichen Gesundheitssystems wird in den USA ein Anteil an Untersuchungen von Patienten zu Hause durchgeführt. Hierdurch werden unnötige Arztbesuche (volle Wartezimmer u. a.) vermieden und Kosten eingespart. Die Abklärung von Patienten zu Hause mit unterschiedlichen Devices hat sich in den USA immer stärker etablieren können. Erst bei auffälligen Befunden zu Hause soll ein entsprechender Arzt aufgesucht werden.

Mit SC wird Männern, die ansonsten einen Arztbesuch eher meiden, die Möglichkeit geboten, eine wenn auch limitierte Abklärung zu Hause durchzuführen. Vor Beginn der klinischen Untersuchung wurde im Rahmen einer Online-Marktanalyse ein sog. „Smoketest“ (explorativer Akzeptanztest) durchgeführt. Hierbei wurde über eine einfache Landingpage das Interesse an einem Heimtestverfahren zur urologischen Basisdiagnostik abgefragt. Innerhalb von 48 h zeigten > 70 % der anonymen Nutzer Interesse an weiterführenden Informationen. Diese Ergebnisse dienten zur Bewertung der potenziellen Akzeptanz und flossen in die Studienplanung ein.

Zu den häufigsten Erkrankungen bei Männern gehören LUTS/BPS, aber auch Nieren- und Blasenerkrankungen. Um einen Überblick über die Blasenfunktion, sowie Nieren- und Blasenerkrankungen zu erhalten, können mit Hilfe des SC der Uroflow, der IPSS-Score und unterschiedliche Biomarker in einer Untersuchung zu Hause bestimmt werden (Blutnachweis im Urin bei u. a. Blasentumoren oder fortgeschrittenem Prostatakrebs; Proteinnachweis bei Nierenerkrankungen und Entzündungen, Nitrit z. B. bei entzündlichen Erkrankungen u. a., Säuremessung über den pH Wert als Risikofaktor für einige Nierensteinleiden).

Bei dem Uroflow handelt es sich auf der einen Seite um eine kostengünstige und gut verfügbare Messmethode. Auf der anderen Seite werden die Messungen von zahlreichen Faktoren wie Patientenalter, Miktionsvolumen, Miktionszeit und Körperhaltung durch die sog. intraindividuelle Variabilität erheblich beeinflusst [8, 11, 22]. Aber auch eine unzureichende Privatsphäre, Unruhe oder Stress in der Praxis oder Klinik erhöhen die intraindividuelle Variabilität und beeinflussen die Ergebnisse [4, 7]. Die Uroflowmessung zu Hause ermöglicht eine Untersuchung in einer geschützten Umgebung, welche die Genauigkeit und Aussagekraft verbessert [3, 4]. Mit der SC-Untersuchung kann ein Uroflow sicher und reproduzierbar, auch zu Hause dargestellt werden (Abb. 3). In der vorliegenden Arbeit wurde bei allen Patienten eine plausible Uroflowkurve dargestellt, das gemessene Urinvolumen wurde ohne wesentliche Abweichung von der Kontrolle korrekt angegeben. Zur Objektivierung der Miktionszeit wurde ein Versuchsaufbau im Labor gewählt. Es zeigte sich, dass das SC-System eine etwas längere Miktionszeit angibt als in der Kontrolle festgestellt wurde. Diese Angaben unterschieden sich nicht signifikant bei einem variablen Flow/Volumen von der Kontrolle. Die maximale Abweichung betrug 4,8 % (Abb. 5). Diese Ergebnisse unterstützen die Zuverlässigkeit des getesteten SC-Systems. Die Biomarker wurden mit einer hohen Sensitivität (100 %) und Spezifität (> 95 %) korrekt erkannt. Vor allem der hohe negative Vorhersagewert unterstützt die Sicherheit der Vorhersage bei den Biomarkern.

Ähnlich der Blutdruckmessung ist der Uroflow v. a. für die longitudinale Beobachtung hilfreich [8]. Auch mit dem SC sind Verlaufsbeobachtungen über die Zeit angedacht. Hierbei soll der Mann ca. einmal pro Monat in den Becher miktionieren. Erst bei auffälligen Abweichungen im Flow, Volumen, Q_max_ oder Miktionszeit wird eine Arztvorstellung empfohlen. Um einen validen Ausgangswert zu erhalten, werden zu Beginn 4 Messungen zu einem Mittelwert zusammengefasst. Bei der Beurteilung der Daten wird auch der IPSS-Fragebogen berücksichtigt. Bei auffälligen Biomarkern erfolgt zunächst eine Kontrolle. Ist auch diese positiv (z. B. wiederholt Hämaturie oder Proteinurie), wird eine Vorstellung beim Facharzt empfohlen. Diese Form der häuslichen Versorgung ersetzt nicht den Gang zum Facharzt bei Beschwerden. Stattdessen soll bei Männern, die sich eigentlich noch „gesund“ fühlen, jedoch pathologische Werte aufweisen, eine Vorstellung und ggf. bessere Therapiemöglichkeit durch frühzeitigere Diagnose ermöglicht werden. Patienten mit unauffälligen Messwerten werden das Wartezimmer nicht unnötig belegen. Patienten mit auffälligen Werten werden motiviert, den Arzt aufzusuchen. Um dem behandelnden Arzt die Ergebnisse übersichtlich darzustellen, werden alle Messungen in einem Medizinreport zusammengefasst. Bei Bedarf können alle Einzelmessungen angesehen werden.

Ein Screening sowohl von Seiten der Biomarker als auch dem Uroflow ist nicht sinnvoll und auch nicht Ziel der SC-Anwendung. Bei auffälligen Befunden ist die weitere Abklärung durch den Urologen mit u. a. Sonographie und Restharnbestimmung aber auch ggf. mit weiteren Laboruntersuchungen und einer Zystoskopie in Abhängigkeit der Befunde erforderlich.

Eine weitere Anwendungsmöglichkeit ist die Therapieüberwachung von z. B. LUTS/BPS-Patienten in der Praxis oder zu Hause. Sollte eine bestimmte Form der bisherigen Therapie ausgereizt sein, kann eine frühzeitige Umstellung erfolgen. Wieweit die Ergebnisse das Patientenverhalten im Hinblick auf eine Arztvorstellung beeinflussen, wird in einer nachfolgenden Studie untersucht.

## Fazit für die Praxis

- Die klinische Untersuchung bestätigt die Leistung, Sicherheit und Benutzerfreundlichkeit des Streamcheck® (SC)-Geräts.
- SC zeigte unter kontrollierten Bedingungen eine konsistente und zuverlässige Messleistung.
- Das System erwies sich über ein breites Altersspektrum hinweg als einsetzbar.
- Die Ergebnisse bestätigen die grundsätzliche Eignung des Geräts für den Heimgebrauch.
- Die hohe Teilnehmerzufriedenheit spricht für eine gute Akzeptanz durch die Anwender.
- Die einfache Handhabung unterstützt den Einsatz im häuslichen Umfeld.
- Die Kombination aus Uroflowmessung und Biomarkeranalyse stellt einen innovativen Ansatz in der urologischen Diagnostik dar.
- Besonders relevant ist die Möglichkeit der Langzeitmessung von Harnflussraten und Biomarkern.
- SC kann dazu beitragen, Veränderungen im Miktionsverhalten und in der Urinzusammensetzung frühzeitig zu erkennen.
- Das System könnte zukünftig mehr Männer zur urologischen Früherkennung bzw. Abklärung motivieren.
- SC stellt damit eine vielversprechende digitale Ergänzung der urologischen Versorgung dar.

## Data Availability

Die erhobenen Datensätze können auf begründete Anfrage in anonymisierter Form beim korrespondierenden Autor angefordert werden. Die Daten befinden sich auf einem Datenspeicher am Universitätsklinikum des Saarlandes.
